# Antimicrobial Treatment Improves Mycobacterial Survival in Nonpermissive Growth Conditions

**DOI:** 10.1128/AAC.02774-13

**Published:** 2014-05

**Authors:** Obolbek Turapov, Simon J. Waddell, Bernard Burke, Sarah Glenn, Asel A. Sarybaeva, Griselda Tudo, Gilles Labesse, Danielle I. Young, Michael Young, Peter W. Andrew, Philip D. Butcher, Martin Cohen-Gonsaud, Galina V. Mukamolova

**Affiliations:** a Department of Infection, Immunity and Inflammation, University of Leicester, Leicester, United Kingdom; b Brighton and Sussex Medical School, University of Sussex, Brighton, United Kingdom; c Medical Microbiology, Centre for Infection and Immunity, Division of Clinical Sciences, St. George's University of London, London, United Kingdom; d Centre de Biochimie Structurale, CNRS UMR 5048, Montpellier, France; e INSERM U1054, Université Montpellier I et II, Montpellier, France; f Institute of Biological, Environmental and Rural Sciences, Aberystwyth University, Aberystwyth, United Kingdom

## Abstract

Antimicrobials targeting cell wall biosynthesis are generally considered inactive against nonreplicating bacteria. Paradoxically, we found that under nonpermissive growth conditions, exposure of Mycobacterium bovis BCG bacilli to such antimicrobials enhanced their survival. We identified a transcriptional regulator, RaaS (for regulator of antimicrobial-assisted survival), encoded by *bcg1279* (*rv1219c*) as being responsible for the observed phenomenon. Induction of this transcriptional regulator resulted in reduced expression of specific ATP-dependent efflux pumps and promoted long-term survival of mycobacteria, while its deletion accelerated bacterial death under nonpermissive growth conditions *in vitro* and during macrophage or mouse infection. These findings have implications for the design of antimicrobial drug combination therapies for persistent infectious diseases, such as tuberculosis.

## INTRODUCTION

The discovery of antimicrobial drugs was a key advance in medical microbiology and has revolutionized the treatment of infectious diseases. The importance of effective antimicrobial drug therapy is highlighted by the current challenges of genotypic drug resistance (1) and phenotypic drug tolerance (2). Antimicrobial agents may either have no effect on bacterial cells (due to drug resistance or tolerance), kill bacteria (bactericidal antimicrobials), inhibit bacterial growth without killing them (bacteriostatic antimicrobials), or, on very rare occasions, support their growth as a nutrient or stabilizer of ribosomes in streptomycin-dependent strains (3, 4). The efficacy of antimicrobials against drug-sensitive bacteria depends on many factors, such as the ability to activate starvation responses (5), cell-cell interactions in heterogeneous populations (6), the host-pathogen interplay (7, 8), and the metabolic state of the cells (9–12). Several infectious diseases, including tuberculosis, are caused by pathogens with the ability to survive in low metabolic activity states, which extends and complicates therapeutic drug regimens.

Tuberculosis remains a leading cause of morbidity and mortality worldwide. Frontline treatment for nonresistant strains includes 6 months of therapy with a combination of four drugs: rifampin, isoniazid, ethambutol, and pyrazinamide. Isoniazid, a prodrug activated by catalase, targets the synthesis of mycolic acids (13). Ethambutol is a bacteriostatic drug that interferes with the synthesis of arabinogalactan and is included in the standard regimen primarily to prevent the emergence of drug resistance (14). Rifampin has a very potent lethal effect on growing and nongrowing Mycobacterium tuberculosis bacilli (15). Finally, pyrazinamide is a prodrug that is converted into pyrazinoic acid (POA) and is capable of killing nonreplicating cells by inhibiting *trans*-translation (16). The potential antimycobacterial drug arsenal has been extended recently by the development of several novel compounds that target cell wall biosynthesis and the respiratory chain (17). In addition, well-known inhibitors of efflux pumps, such as verapamil and reserpine, are now considered possible chemotherapeutic agents (8, 18). However, elimination of persistent or nonreplicating M. tuberculosis bacilli still presents serious challenges, mainly due to our limited knowledge of the mechanisms underlying their transition to nongrowing states. M. tuberculosis is able to survive *in vivo* and *in vitro* for years, as evidenced by the observation that one-third of the global population is estimated to be latently infected with this bacterium (19, 20). Moreover, experimental data suggest that M. tuberculosis can survive stasis efficiently and, unlike many other bacteria, retains high viability through the stationary phase (12) and in chronic infection models (21, 22). Numerous studies have identified and described factors that mediate successful M. tuberculosis survival in the stationary phase. These factors include enzymes involved in specific metabolic adaptations, transcriptional regulators, sigma factors, stress response proteins, and cell wall enzymes (12).

In the present study, we further reveal the complexity of metabolic regulation in mycobacteria during their transition to a nonreplicating state. Our data suggest that tight control of efflux pumps is critical for bacterial survival in nongrowing conditions. Moreover, we show how certain frontline antimicrobials may influence this control and actually improve bacterial survival under nonpermissive growth conditions. These findings offer an alternative strategy for targeting nonreplicating bacilli *in vivo*, precisely the bacteria that are most difficult to eliminate with current antimicrobials (15, 23–25).

## MATERIALS AND METHODS

### Organisms and media.

Mycobacterium bovis BCG Glaxo strain and Mycobacterium tuberculosis H37Rv were grown in Sauton's or Middlebrook 7H9 liquid medium (Becton, Dickinson and Company) supplemented with albumin-dextrose complex. For generation of prolonged stationary phase, 2 μl from a 1-month-old culture was inoculated in 20 ml of supplemented Sauton's medium (the composition of this medium is described in the supplemental material) in 100-ml flasks sealed with Suba-Seal stoppers (William Freeman Ltd., Barnsley, United Kingdom). The inoculated flasks were incubated at 37°C without shaking. Chemicals were added 30 days after inoculation at the following final concentrations (in μg/ml): ethambutol, 20; isoniazid, 50; cerulenin, 50; streptomycin, 100; metronidazole, 50; reserpine, 20; verapamil, 20; carbonyl cyanide *m*-chlorophenylhydrazone (CCCP), 10. Sterile water (or dimethyl sulfoxide [DMSO]) was added to control cultures. Viability was assayed by estimation of CFU and most probable number (MPN) counts as described previously (24). Briefly, for CFU counts, 10-μl drops of serially diluted bacteria were spotted on 7H10 agar; for MPN counts, 50-μl aliquots of serially diluted bacteria were inoculated into 48-well microtiter plates containing 450 μl of supplemented Sauton's medium diluted with culture supernatant obtained from a growing M. bovis BCG culture (optical density at 580 nm [OD_580_], 0.8). Bacterial suspensions were passed through a 23-gauge needle to break aggregates. For MPN and CFU counts, 4 to 8 replicates of each dilution were inoculated in supplemented Sauton's medium or on 7H10 agar plates. The inoculated plates were sealed with Nescofilm, placed in plastic bags, and incubated at 37°C for 6 weeks without shaking. MPN counts were determined using a published protocol. MPN counts were calculated with 95% confidence limits by using the FDA's procedure (24). Data are presented as the percent survival relative to that of the stationary-phase culture before treatment (means ± standard errors of the means [SEM], *n* > 3). All experiments were repeated at least 3 times.

### *raaS* overexpression and deletion.

The RaaS-encoding region, including 31 bp upstream of the predicted coding sequence, was amplified from M. tuberculosis H37Rv DNA and cloned into the BamHI and SpeI sites of the pMind plasmid (26). *raaS* overexpression was induced by the addition of 20 ng/ml tetracycline. Overexpression of *raaS* was confirmed by quantitative reverse transcription-PCR (qRT-PCR). In-frame 573-bp *raaS* deletion mutants of M. tuberculosis H37Rv and M. bovis BCG were generated by using a homologous recombination approach as described previously (27). Deletion mutants were confirmed by PCR and sequencing. To complement the knockout mutants, full-length *rv1219c* (*raaS*_Mtb_) was cloned into SpeI and HpaI sites of an integrating vector, pRBexint, to ensure constitutive expression from a *dnaK* promoter (28). All oligonucleotides used in this study are detailed in Table S2 of the supplemental material.

### Transcriptional profiling.

Total RNA was isolated from 30-ml aliquots from M. bovis BCG stationary-phase cultures after 24 h of exposure to the antimicrobial compounds, using the TRIzol method (29) (for details, see the supplemental material). Microarray data were confirmed by quantitative RT-PCR.

### Purification of recombinant RaaS.

The *raaS* gene was cloned into NdeI and NheI sites of the pET15-Tev plasmid to generate a 6×His-tagged recombinant protein. Protein expression was induced by isopropylthio-β-galactoside at a final concentration of 0.2 mM. Recombinant RaaS was purified by using a HiTrap 1-ml immobilized-metal affinity high-performance column (Amersham Biosciences). The structural integrity of the RaaS protein was validated by circular dichroism.

### Electrophoretic mobility shift assay.

The RaaS upstream region, or synthetic oligonucleotides covering the upstream region, was used for the experiments. DNA was mixed with purified RaaS protein in buffer containing 25 mM Tris (pH 8.0), 50 mM NaCl, 0.25 mM EDTA. The mixture was run on a 5% (wt/vol) polyacrylamide gel in 0.25× Tris-borate-EDTA buffer. Ethidium bromide-stained gels were visualized and the images captured using the GeneSnap system (Syngene UK). In some experiments, shifted bands were visualized using ^32^P-radiolabeled double-stranded oligonucleotides.

### Fluorescence anisotropy.

The synthetic oligonucleotides, containing imperfect direct repeats, were covalently labeled with Atto 647N succinimidyl ester dye (Invitrogen). Steady-state fluorescence anisotropy binding titrations were carried out on a Tecan Safire II microplate reader, using a 635-nm light-emitting diode for excitation and a monochromator set at 680 nm (bandwidth, 20 nm) for emission in buffer containing 50 mM Tris-HCl (pH 8.5) and 150 mM NaCl.

### Macrophage infection experiments.

The human Mono Mac 6 macrophage-like cell line (30) was infected with M. tuberculosis bacilli at a multiplicity of infection (MOI) of 5 or 0.1. Infection and MOIs were confirmed by staining 2% (wt/vol) paraformaldehyde-fixed macrophages by using the TB Quick kit (Reactif RAL). For CFU counts, infected macrophages were lysed with 0.006% (wt/vol) SDS at 24, 48, 72, 96, and 120 h postinfection, serially diluted, and plated on 7H10 plates. Three independent experiments were done for each MOI.

### Mouse infection experiments.

M. bovis BCG Glaxo cells (wild type [WT], the Δ*raaS* strain, and the corresponding *raaS*-complemented strain, Δ*raaS*_com_) were grown in 7H9 supplemented medium to logarithmic phase (OD_580_, 0.4 to 0.6), washed three times with 10% (vol/vol) glycerol, and flash-frozen in liquid nitrogen. Before infection, defrosted cells were centrifuged to remove glycerol; the cell pellets were resuspended in phosphate-buffered saline and passed through a 25-gauge needle 10 times. BALB/c mice (Charles River, United Kingdom) were lightly anesthetized with 2.5% (vol/vol) isoflurane over oxygen (1.8 to 2 liters min^−1^) and infected intranasally with 1 × 10^6^ mycobacteria. For each time point, bacterial loads from 5 separate mice were determined. At 1, 7, 10, 14, 17, and 21 days postinfection, mice were culled and their lungs and spleens homogenized in 7H9 medium for determination of CFU counts. Serially diluted cells were plated on 7H10 agar, and plates were incubated 37°C for up to 8 weeks.

### Statistical analysis.

All data are presented as means ± SEM (*n* > 3). For statistical analysis of bacterial survival *in vitro*, an unpaired *t* test was employed. Statistically significant differences in bacterial loads in mice were determined by a one-way analysis of variance (ANOVA). Significantly differentially expressed genes were identified by comparing the results with the knockout mutant to both wild-type and complemented strain results by using a *t* test (at a *P* level of <0.05, with Benjamini and Hochberg multiple testing correction) and a change threshold of >2-fold.

### Ethics statement.

Experiments were carried out in accordance with the Animals (Scientific Procedures) 1986 protocols and under Home Office project license number 60/4327. Experiments were approved by the Research Ethics Committee, University of Leicester, Leicester, United Kingdom.

### Microarray data accession numbers.

Fully annotated microarray data have been deposited in the BμG@Sbase database (accession number E-BUGS-123 [http://bugs.sgul.ac.uk/E-BUGS-123]) and also ArrayExpress database (accession number E-BUGS-123).

## RESULTS

### Antimicrobials enhance bacterial survival under nonpermissive growth conditions.

**FIG 1:**
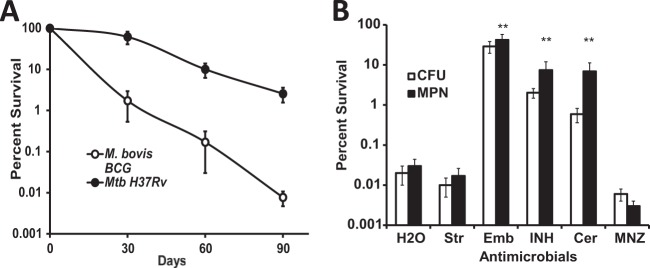
Survival of M. bovis BCG in prolonged stationary phase and the effect of antimicrobial treatment. (A) M. bovis BCG and M. tuberculosis were grown in sealed flasks to stationary phase and were incubated for a further 90 days. (B) One-month-old M. bovis BCG cultures were treated with antimicrobials and incubated for an additional 2 months before viability was assayed. **, statistically different from untreated control (*P* < 0.01; *n* = 5). H_2_O, water control; Str, streptomycin; Emb, ethambutol; INH, isoniazid; Cer, cerulenin; MNZ, metronidazole. Mean values of five independent experiments are presented.

We first compared survival of M. tuberculosis H37Rv and M. bovis BCG in prolonged stationary phase in sealed flasks without shaking. While a reduction in viable bacterial counts was observed for both organisms, the survival dynamics of M. tuberculosis and M. bovis BCG populations differed (Fig. 1A). A 3-month incubation resulted in a 1,000-fold difference in survival between M. tuberculosis and M. bovis BCG cultures, confirming that M. bovis BCG is attenuated for long-term persistence *in vitro* compared to M. tuberculosis (*P* < 0.001, *t* test). To establish whether stationary-phase M. bovis BCG populations have a mixture of dying and slowly growing cells, we treated 1-month-old M. bovis BCG cultures with antimicrobials with activity against replicating bacteria, targeting cell wall biosynthesis (ethambutol, isoniazid, and cerulenin) or inhibiting the translation machinery (streptomycin). To avoid selection of resistant mutants and ensure the presence of active antimicrobials during the prolonged exposure, we applied very high concentrations of antimicrobials that greatly exceeded the MICs. The effects of these drugs on cell viability were followed over a 2-month period. After 24 h of exposure, streptomycin caused marked cell death (>2 log_10_) of the 1-month-old cultures, with cell survival of 0.23% ± 0.1%. Ethambutol, isoniazid, or cerulenin had no effect on cell viability (see Fig. S1 in the supplemental material), indicating that the cells were probably not actively replicating and not undergoing cell wall synthesis. As described above, a 2-month incubation of drug-free control cultures resulted in a pronounced loss of viability from ca. 10^7^ cells/ml to ca. 10^4^ cells/ml, while treatment with streptomycin and metronidazole reduced viable counts to ca. 10^3^ cells/ml (Fig. 1B). In contrast, cultures incubated for 2 months in the continuous presence of antimicrobial compounds affecting cell wall biosynthesis still contained substantial numbers of viable cells (>10^6^ viable cells/ml). In the case of ethambutol, more than 40% of the population remained viable, compared with 0.02% in the drug-free control (Fig. 1B). Interestingly, the posttreatment lag phase upon resuscitation of growth in fresh drug-free liquid and solid media was reduced in ethambutol-treated bacilli (20 ± 2 days for the growth of visible colonies) relative to untreated bacilli (30 ± 4 days), indicating that the treated cells were probably less damaged during storage and did not require additional time for recovery. Importantly, surviving M. bovis BCG bacilli remained fully sensitive to the drugs used, with identical MICs for treated and untreated cells (see the MIC determinations reported in the supplemental material); therefore, emergence of genotypic resistance may be excluded as a mechanism of bacterial survival in drug-treated samples.

This antimicrobial-assisted improvement of bacterial survival in M. bovis BCG cultures was only observed in sealed flasks that were not shaken. It was highly growth phase dependent, occurring only after stationary phase had been reached and not during log-phase growth, indicating the importance of nonpermissive growth conditions for this persistence phenomenon. We next focused our efforts on elucidating the mechanisms underlying the observed survival defect of M. bovis BCG and its improvement by antimicrobial treatment.

### The predicted transcriptional regulator RaaS is important for antimicrobial-enhanced survival under nonreplicating conditions.

**FIG 2:**
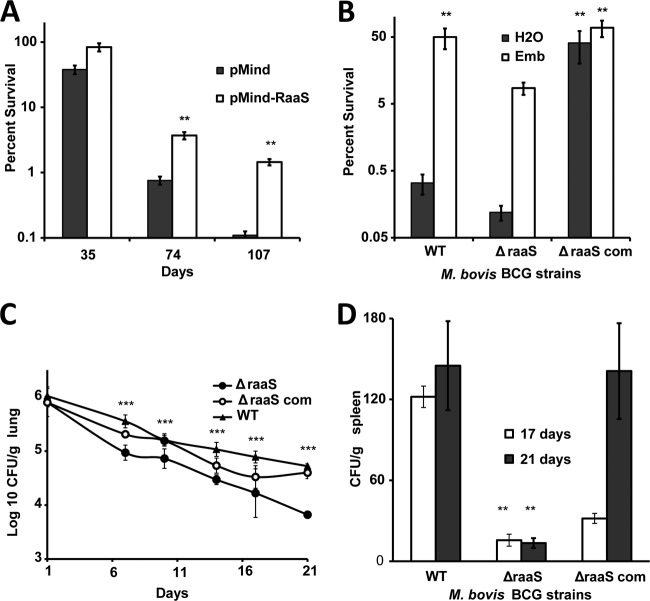
Effects of *raaS* overexpression or deletion on survival of M. bovis BCG in nonpermissive growth conditions *in vitro* and *in vivo*. (A) Strains containing pMind or pMind-*raaS* were incubated in tetracycline-supplemented medium for 107 days. **, statistically different survival between pMind-*raaS* and pMind (*P* < 0.01; *n* = 3). (B) Stationary-phase cultures of wild-type, Δ*raaS*, and Δ*raaS*_com_ strains were treated with ethambutol and incubated for a further 2 months. **, statistically significant enhanced survival compared to drug-free wild-type control (*P* < 0.01; *n* = 3). (C and D) Bacterial loads in lungs (C) and spleens (D) of infected mice. (C) ***, Δ*raaS* strain loads in lungs at 7 to 21 days were statistically lower than wild-type or Δ*raaS*_com_ strain loads (*P* < 0.001). (D) **, Δ*raaS* strain loads in spleens were statistically significantly lower (*P* < 0.01) than wild-type and Δ*raaS*_com_ strain loads.

Whole-genome transcriptional profiling of 1-month-old M. bovis BCG cultures revealed a common signature of differentially expressed genes for ethambutol-, cerulenin-, or isoniazid-treated bacilli compared with streptomycin-treated or drug-free bacteria (see Table S1 and Fig. S2 in the supplemental material). Of particular interest among these genes, *bcg_1279c* encodes a predicted transcriptional regulator of unknown function, with orthologues in M. tuberculosis (*rv1219c*) and M. bovis (*mb1251c*). In M. bovis BCG Glaxo and Pasteur strain, Bcg_1279c (which we designated RaaS, for regulator of antimicrobial-assisted survival, based on the findings described below) only differs in its M. tuberculosis and M. bovis homologues by one amino acid (W113C), due to a single G-to-C substitution. Both proteins are likely to have similar roles in these closely related organisms. We reasoned that the induction of this transcriptional regulator 24 h after treatment with cell wall-targeting antimicrobials might be responsible for controlling an adaptive response that enhances the survival of mycobacteria in extended stationary phase. We cloned and overexpressed *M. tuberculosis raaS* (*raaS*_Mtb_) in M. bovis BCG under the control of a tetracycline-inducible promoter. Quantitative RT-PCR confirmed that tetracycline treatment induced *raaS* expression 25-fold. Mycobacteria overexpressing *raaS*_Mtb_ survived better in prolonged stationary phase (Fig. 2A) and recovered sooner than the empty vector control (see Fig. S3 in the supplemental material), highlighting a role for RaaS in long-term mycobacterial viability.

The functional significance of RaaS in antimicrobial-assisted survival was further explored by constructing an M. bovis BCG *raaS* in-frame deletion mutant (Δ*raaS*), and the corresponding *raaS*-complemented strain (Δ*raaS*_com_) (Fig. 2B). The Δ*raaS* mutant did not display any significant defects in the logarithmic growth phase (see Fig. S4 in the supplemental material), confirming the previous observation from screening of an M. tuberculosis H37Rv transposon library that defined *raaS* as nonessential for mycobacterial growth *in vitro* (31). The survival of the Δ*raaS* strain was reduced compared to the wild type during a drug-free extended stationary phase, with only 0.12% and 0.33%, respectively, of initial populations recovered (Fig. 2B). The knockout mutant complemented with wild-type *raaS* on an integrating vector, the Δ*raaS*_com_ strain, survived long-term stationary phase to a much greater degree, with 41% of the original population able to produce colonies on solid medium. This phenotype was observed in the Δ*raaS* mutant strains complemented either by M. tuberculosis or M. bovis BCG versions of *raaS* (survival values in both strains did not differ statistically [*P* > 0.1, *t* test]), confirming that RaaS from either bacterium is capable of playing the same role. This dramatic increase in survival (without the addition of drug) in the complemented strain compared to the wild type is likely to be due to the overexpression of *raaS* in the complemented strain, analogous to the effect observed when *raaS* was deliberately overexpressed (Fig. 2A). Quantitative RT-PCR demonstrated that *raaS* was indeed 2.6-fold overexpressed in the complemented strain compared to the wild type. The addition of ethambutol after 1 month of incubation was beneficial for the survival of all strains, with approximately 50% of the original populations of wild-type and complemented M. bovis BCG bacilli viable after incubation for a further 2 months (Fig. 2B). Only 8% of the Δ*raaS* strain population remained viable. This confirmed that deletion of *raaS* limited the ability of mycobacteria to survive after long-term antimicrobial exposure and also indicated that additional factors might be involved in regulation of this pathway.

To investigate whether RaaS plays a role in mycobacterial survival *in vivo*, three separate groups of BALB/c mice were infected with either wild-type M. bovis BCG, the *raaS* deletion mutant strain (Δ*raaS*), or the Δ*raaS* complemented mutant strain (Δ*raaS*_com_). M. bovis BCG does not replicate in BALB/c mice, and after several weeks of infection most bacilli are cleared from murine lungs (32, 33). Therefore, this model may be considered an *in vivo* model of bacterial survival under conditions nonpermissive for growth. Figure 2C shows the fate of M. bovis BCG in mouse lungs during 3 weeks of infection. A decrease in bacterial numbers was observed in all strains. However, the deletion mutant strain was attenuated compared to the wild type and the complemented mutant strain (*P* < 0.001, one-way ANOVA). After 3 weeks, there was a 10-fold difference in bacterial loads of the Δ*raaS* strain and the wild type in the lungs (Fig. 2C); complementation of the deletion restored the wild-type phenotype. In murine spleens, BCG could only be detected at 17 and 21 days because of gradual dissemination from the lungs (Fig. 2D). The mutant phenotype in the spleen was partially restored at 17 days and fully complemented at 21 days. Thus, our data demonstrate that RaaS is functionally significant both *in vitro* and *in vivo*.

### RaaS acts as a transcription repressor of predicted ATP-dependent efflux pumps.

**FIG 3:**
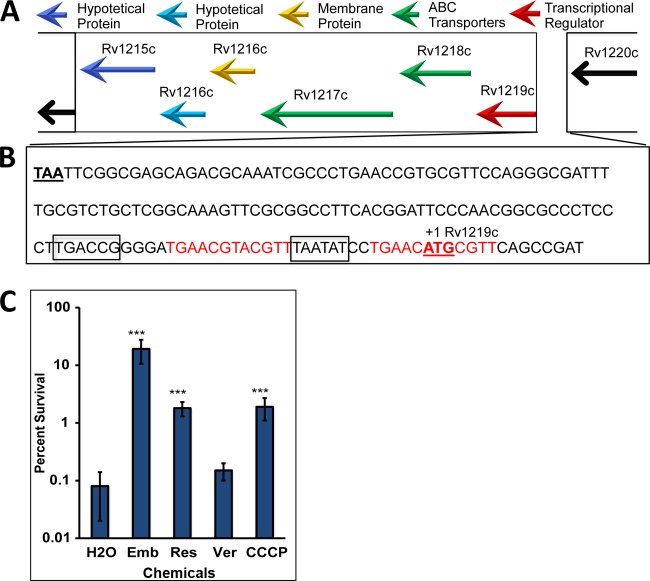
RaaS binds to its upstream region. (A) Schematic representation of the *raaS* operon in M. tuberculosis. (B) Intergenic region between *raaS* and *rv1220c*, containing the predicted −10 and −35 promoter elements (boxed). The RaaS-binding site is shown in red; the predicted *raaS* start and *bcg1280c* stop codons are underlined. (C) Influence of efflux inhibitors on survival of M. bovis BCG in extended stationary phase. ***, ethambutol (Emb)-, reserpine (Res)-, or CCCP-treated cells survived significantly better (*P* < 0.001) than drug-free controls.

To identify the RaaS regulon, we performed microarray-based whole-genome transcriptional profiling of the Δ*raaS* mutant strain relative to the complemented mutant strain and wild-type M. bovis BCG. Seven genes were significantly differentially expressed in the Δ*raaS* mutant strain compared to either the wild-type or complemented mutant strains (Table 1). This pairwise comparison controlled for polar effects that might have resulted from the removal of *raaS* from the knockout mutant. As an additional precaution, the sequence of this region of the genome in the knockout and complemented strains was confirmed to be identical (other than the deletion of *raaS*). Of the putative RaaS regulon (Table 1), one gene (*bcg3553*, homologous to *rv3489*) was repressed in the Δ*raaS* strain, while 5 genes (*drrC* and *bcg1278c/bcg1277c/bcg1276c/bcg1275c*, corresponding to *rv1218c*/*rv1217c/rv1216c/rv1215c*) were induced. This indicated that RaaS likely acts as a repressor of *drrC* (*bcg2960*), which encodes an annotated efflux pump, as well as the cluster of genes located immediately downstream from itself (Fig. 3A and B). These genes are predicted to encode ABC transporters (*bcg1278c*/*bcg1277c*) and are associated with integral membrane proteins (*bcg1276c*/*bcg1275c*), suggesting that downregulation of the cellular export machinery is required for mycobacterial survival under conditions nonpermissive for growth. Interestingly, only *bcg1278c* and *raaS* were among the genes differentially expressed in M. bovis BCG treated with the antimicrobials that improved bacterial survival compared to the control (see Table S1 in the supplemental material). Our results strongly suggest that antimicrobial-mediated activation of this transcriptional regulator has a protective effect in extended stationary phase, by repressing the expression of putative efflux pumps. In accordance with this conclusion, an increase in viability, compared to the control was observed in extended stationary phase after exposure of 1-month-old M. bovis BCG cultures to the ABC efflux pump inhibitor reserpine or the proton ionophore CCCP (Fig. 3C). Verapamil had a very moderate effect on bacterial viability, presumably due to its relatively low stability in solutions with pH over 6.0 (34).

**TABLE 1:** Genes significantly differentially expressed in the Δ*raaS* strain versus the WT or Δ*raa*S_com_ strain, thus defining the putative RaaS regulon

M. bovis BCG gene	M. tuberculosis homologue	Predicted function	Fold change for Δ*raaS* strain vs WT or Δ*raaS*_com_ strain (assay method)^*a*^				Fold change for Δ*raaS*_com_ vs WT (qRT-PCR)
			WT (MA)	Δ*raaS*_com_ (MA)	WT (qRT-PCR)	Δ*raaS*_com_ (qRT-PCR)	
*bcg1278c*	*rv1218c*	ABC transporter	29.56	46.45	48.9	82.79	−1.69
*bcg1277c*	*rv1217c*	ABC transporter	21.14	34.86	33.00	62.95	−1.81
*bcg1276c*	*rv1216c*	Methyltransferase	8.13	12.10	13.97	25.37	−1.81
*bcg1275c*	*rv1215c*	Peptidase	2.50	3.00	2.15	1.95	1.09
*drrC*	*drrC*	ABC transporter	2.07	2.09	2.00	1.26	1.59
*bcg3553*	*rv3489*	Hypothetical protein	−2.22	3.45	−1.05	−1.22	1.15

a Fold change relative to the WT or Δ*raaS*_com_ strain was determined by microarray (MA) or qRT-PCR.

### Identification and characterization of the *RaaS* DNA binding site.

**FIG 4:**
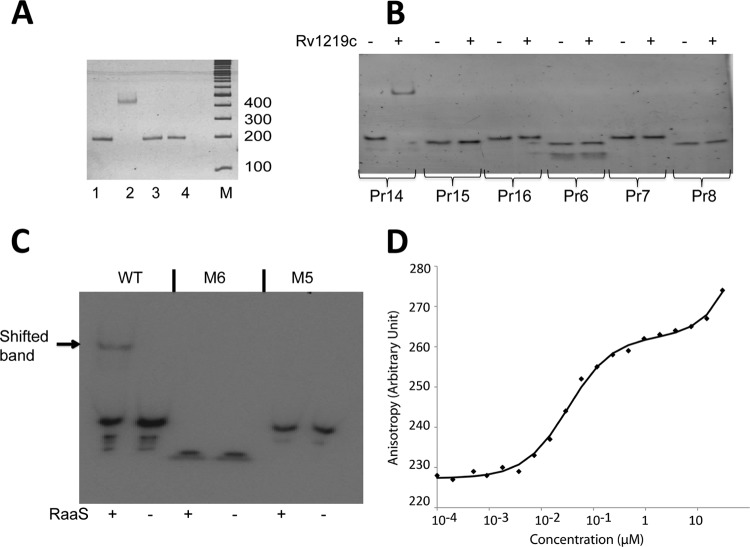
Characterization of the RaaS-binding site. (A) Electrophoretic mobility shift assay results. A 174-bp DNA fragment (the *raaS* upstream region) was mixed with proteins. Lanes 1, DNA alone; 2, DNA with RaaS; 3, DNA with bovine serum albumin; 4, DNA with rPASTA protein; M, DNA markers. (B) Identification of the RaaS-binding site. Six pairs of oligonucleotides (Pr6, -7, -8, -14, -15, and -16 [for the sequences, see Table S2 in the supplemental material]), covering the *raaS* upstream region, were used in binding experiments with RaaS. (C) ^32^P-labeled annealed oligonucleotides (20 nM) were mixed with 170 nM purified RaaS. WT is Pr14 (GGGATGAACGTACGTTTAATATCCTGAACATGCGTTCAG); M6 is a single-repeat oligonucleotide (GGGTGAACATGCGTTCA), and the mutated double-repeat oligonucleotide is M5 (GGGATGAACGTACG**AAA**AATATCCTGAACATGCGTTCAG). In the M5 oligonucleotide, 3 thymidines were replaced with adenosines (underlined) to interfere with possible DNA secondary structures. (D) Anisotropy binding profile for interaction of Atto647N-DNA (4 nM) with RaaS.

*raaS* is the first gene in an operon containing 5 genes (Fig. 3A) and is separated by 140 nucleotides from the upstream gene *bcg1280c* in *M. tuberculosis rv1220c* (Fig. 3A and B). The nucleotide sequence of this intergenic region is identical in M. bovis BCG and M. tuberculosis. Electrophoretic mobility shift assays revealed that RaaS was able to bind to this region (Fig. 4A). To identify a binding site, we designed a series of oligonucleotides covering the entire region between *bcg1280c* and the first 10 nucleotides of *raaS* (Fig. 3B) and performed electrophoretic mobility shift assays (Fig. 4B). We found that only oligonucleotides containing two 12-bp imperfect direct repeats separated by the predicted *raaS* −10 promoter element were bound by RaaS (Fig. 3B and 4B). Both repeats were necessary for RaaS binding, as truncation of the sequence or replacement of three thymidines in one repeat with adenosines led to complete loss of binding (Fig. 4C). Each repeat was characterized by the presence of two 5-nucleotide inverted repeats, and the second repeat included the predicted start codon of *raaS* (Fig. 3B). Such organization of the operon suggests that RaaS represses expression of its own gene and other genes in the operon, supporting our regulon prediction from the transcriptomics signatures. Moreover, the necessity of both imperfect direct repeats for RaaS binding suggests that RaaS interacts with the DNA sequence, as shown for transcriptional regulators (35).

The specific binding of RaaS to the identified DNA site was further confirmed by fluorescence anisotropy. Oligonucleotides defining the binding site were labeled with Alexa Fluor 488 dye and mixed with unlabeled RaaS. RaaS exhibited a strong specific DNA-binding activity with an estimated binding constant of 31 nM ± 6 nM (Fig. 4D). Thus, RaaS can be classified as an autotranscriptional repressor. An autotranscriptional repressor, encoded by the first gene in an operon, inhibits transcription of itself and the other genes in the operon. Such an organization ensures a tight regulation of operon expression, and autotranscriptional repressors are highly conserved in bacteria (36).

### RaaS is important for survival in prolonged stationary phase and during macrophage infection.

**FIG 5:**
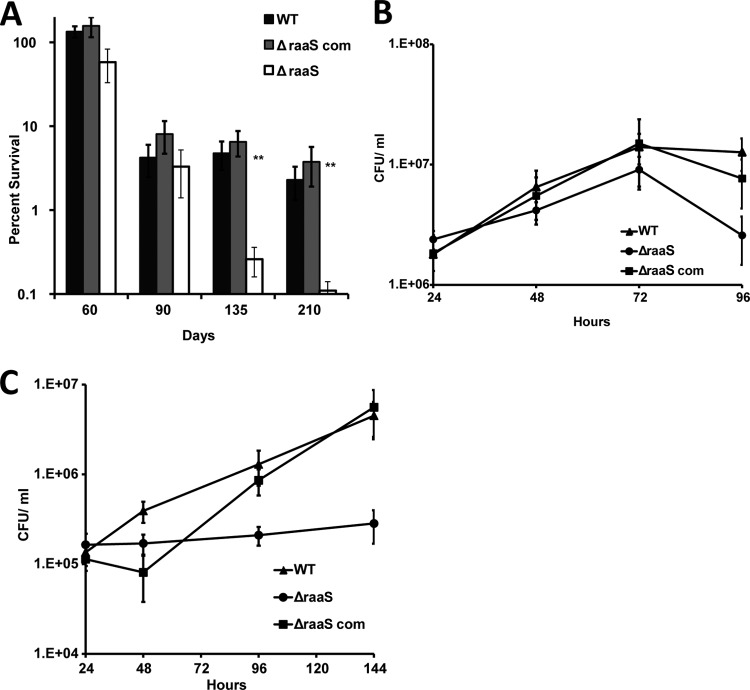
RaaS is important for persistence of M. tuberculosis in prolonged stationary phase and in human macrophages. (A) Wild-type, Δ*raaS*_Mtb_, and Δ*raaS*_Mtb_ com strains were incubated in 7H9 medium without shaking at 37°C for 7 months. **, Δ*raaS*_Mtb_ had a significant survival defect (*P* < 0.01; *n* = 3) compared to the wild-type or Δ*raaS*_Mtb_ com strains after 135 and 210 days of incubation. (B and C) Mono Mac 6 cells were infected with wild-type, Δ*raaS*_Mtb_, and Δ*raaS*_Mtb_ com strain cells at an MOI of 5 (B) or 0.1 (C). The Δ*raaS*_Mtb_ strain had a significant survival defect (*P* < 0.01; *n* = 3) compared to the wild-type or Δ*raaS*_Mtb_ com strain after 96 and 144 h.

Finally, we investigated whether RaaS contributed to persistence of M. tuberculosis in prolonged stationary phase *in vitro* and during macrophage infection. In our initial experiments, we attempted to study the effect of ethambutol and reserpine on survival of M. tuberculosis in stationary phase. However, as mentioned above, unlike M. bovis BCG, M. tuberculosis retained a relatively high level of viability after 2 months of incubation in stationary phase. Nearly 30% ± 5% of bacteria could be recovered from control flasks, while ethambutol- or reserpine-treated cultures contained even higher numbers of viable mycobacteria, 45% ± 6% and 67% ± 10%, respectively. The differences between control and both treated samples were statistically different (*P* < 0.001, *t* test); however, they were not as marked as seen in the case of M. bovis BCG. Further incubation is likely to have resulted in degradation of drugs; therefore, we were not able to demonstrate that the drugs had similar effects on M. tuberculosis as on M. bovis BCG. We therefore investigated the role of RaaS in survival of untreated M. tuberculosis for longer periods. The viability of the *M. tuberculosis raaS* knockout mutant (Δ*raaS*_Mtb_) decreased with incubation time (in drug-free medium) compared to the wild-type and Δ*raaS*_Mtb_-complemented (Δ*raaS*_Mtb_ com) strains, with approximately 20 times fewer viable Δ*raaS*_Mtb_ bacteria than the wild type after 7 months of culture (Fig. 5A). Complementation of the mutant fully restored the wild-type phenotype (Fig. 5A). Loss of RaaS function also impacted M. tuberculosis intercellular survival. At an MOI of 5, Δ*raaS*_Mtb_ bacilli were able to multiply similarly to the wild type in the human macrophage Mono Mac 6 cell line in the first 72 h; however, survival was reduced in the late stages of infection (*P* < 0.01), indicating that RaaS plays an important role in long-term survival of M. tuberculosis in macrophages (Fig. 5B). This survival defect was abolished in the complemented mutant. At a lower MOI (0.1), the Δ*raaS*_Mtb_ strain was not able to replicate in macrophages, in contrast to the wild type (Fig. 5C), while complementation of the mutant restored the wild-type phenotype. Our results confirmed that RaaS is required for successful persistence of *M. tuberculosis in vitro* and in macrophages. Additionally, we established that *raaS* was 3.65-fold (±0.16-fold) overexpressed in M. tuberculosis compared to M. bovis BCG, suggesting that downregulation of *raaS* in the latter species may contribute to its impaired survival in stationary phase.

## DISCUSSION

There is an increasing understanding of the complexity of bacterial physiological processes that, as in eukaryotic cells, are influenced by periods of nongrowth, senescence, and death. Here we have proposed a molecular mechanism for regulating efflux that contributes to mycobacterial survival under nonpermissive growth conditions. We first presented data showing that M. bovis BCG has a general survival defect under nonreplicating conditions (Fig. 1A), which was significantly improved on administration of several antimicrobial compounds targeting cell wall biosynthesis (Fig. 1B). We identified a transcriptional regulator (Rv1219c, named RaaS in this study) that was strongly induced by the antimicrobial treatment and mediated expression of the putative ATP-dependent efflux pump encoded by *bcg1278c/bcg1277c* (*rv1218c/rv1217c*). Whereas overexpression of *raaS* from two different plasmid systems mimicked the survival-promoting effect of ethambutol, *raaS* deletion substantially reduced the ethambutol effect on mycobacterial survival. However, it did not abolish the ethambutol effect completely, indicating that other factors might be involved in the regulation of this pathway. Indeed, ChIP-seq data maps (available at the TB Database [http://genome.tbdb.org/]) show the binding of at least two additional transcription factors (Rv0880 and Rv2779c) to the RaaS operon that may partially compensate for the loss of RaaS under certain conditions. Nevertheless, our data on overexpression of *raaS* in M. bovis BCG strongly support the proposed role of RaaS as a major regulator of metabolite-regulated shutdown of efflux and consequent improvement of mycobacterial survival. Why does downregulation of efflux stimulate mycobacterial survival? It has been proposed that efflux pumps are involved in general cellular detoxification, and therefore they could be important in nonreplicating persistence (37). We argue, however, that the toxic by-products of active metabolism might be reduced in conditions of low nutrient availability. Indeed, reduced membrane transport processes and low metabolic activity are considered important characteristics of nongrowing persistent bacteria (10, 38, 39). Therefore, shutdown of efflux to minimize the use of ATP and the loss of potential metabolites would be beneficial for nongrowing bacteria, but it would require complex regulatory cascades to control these processes. Although we do not yet know precisely how exposure to antimicrobial compounds triggers RaaS-mediated shutdown of the Rv1218c-Rv1217c efflux pump, our findings suggest that ATP-dependent pumps are an important part of the metabolic circuitry and energy metabolism of growing cells, as their activity depends on the metabolic state of the bacterium and is finely tuned by metabolites binding to transcriptional regulators. We hypothesize that RaaS expression is regulated by metabolites produced during active growth. Under stressful conditions (for example, hypoxia or iron limitation), mycobacteria slow their metabolism and redirect metabolic fluxes from the tricarboxylic cycle to alternative pathways and to the synthesis of storage compounds (40, 41). In stressful conditions, free RaaS binds to its DNA recognition sequence, repressing the transcription of this cluster of efflux pump genes and also itself, in a classical feedback loop. We propose that antimicrobial treatment potentiates this process, directly or indirectly influencing levels of the putative RaaS ligand. The presented results indicate that RaaS is a part of the complex regulatory mechanisms that orchestrate a coordinated downregulation of energy-consuming processes and the activation of long-term persistence. This hypothesis is supported by our findings that M. tuberculosis employs the RaaS-mediated mechanism for *in vitro* persistence during *in vivo* macrophage infection and in prolonged stationary phase (Fig. 5A to C). Our results on *raaS* upregulation in M. tuberculosis indicate that this RaaS-mediated mechanism is partially disabled in M. bovis BCG compared with M. tuberculosis, which contributes to the failure of M. bovis BCG to persist in growth nonpermissive conditions. Overexpression of *rv1219c* in M. bovis BCG overcomes this defect.

Our results led us to two clinically relevant conclusions. Uncontrolled expression of a single ABC transporter can impair the viability of nonreplicating mycobacteria and therefore may be used as a strategy to kill them. An argument against this therapeutic approach is that excessive expression of an efflux pump may result in multiple-drug resistance of mycobacteria. However, alteration of Rv1218c efflux pump expression does not influence sensitivity to frontline antimicrobials (42). Therefore, dysregulation of this efflux system by using low-molecular-weight compounds to disrupt the DNA-binding activity of RaaS, analogous to inhibition of the transcriptional regulator EthR (43), might offer a novel opportunity to specifically target persistent M. tuberculosis bacilli. Our results suggest that efflux pump inhibitors, which have recently been recognized as a new rich source of drugs for the prevention of multiple-drug resistance in replicating bacteria (8, 18), should be used with caution for the treatment of infections associated with nonreplicating bacteria, as they may actually promote the long-term survival of the bacteria.

## Supplementary Material

Supplemental material
