# Supplementary material for: Antimicrobial Treatment Improves Mycobacterial Survival in Nonpermissive Growth Conditions
Source: Antimicrob Agents Chemother. 2014 May;58(5):2798–806. doi: 10.1128/AAC.02774-13 (PMC3993263; doi:10.1128/AAC.02774-13)
Supplement: Supplemental material [file supp_58_5_2798__index.html]

Antimicrobial Treatment Improves Mycobacterial Survival in Nonpermissive Growth Conditions — Supplemental material 

# Antimicrobial Treatment Improves Mycobacterial Survival in Nonpermissive Growth Conditions

## Supplemental material

**Files in this Data Supplement:**

- Supplemental file 1 -

  Supplemental Figures S1 to S4, additional experimental details, and Tables S1 and S2.

  PDF, 629K
