# Supplementary material for: Antimicrobial Treatment Improves Mycobacterial Survival in Nonpermissive Growth Conditions
Source: Antimicrob Agents Chemother. 2014 May;58(5):2798–806. doi: 10.1128/AAC.02774-13 (PMC3993263; doi:10.1128/AAC.02774-13)
Supplement: Supplemental material [file AAC.02774-13_zac005142838so1.pdf]

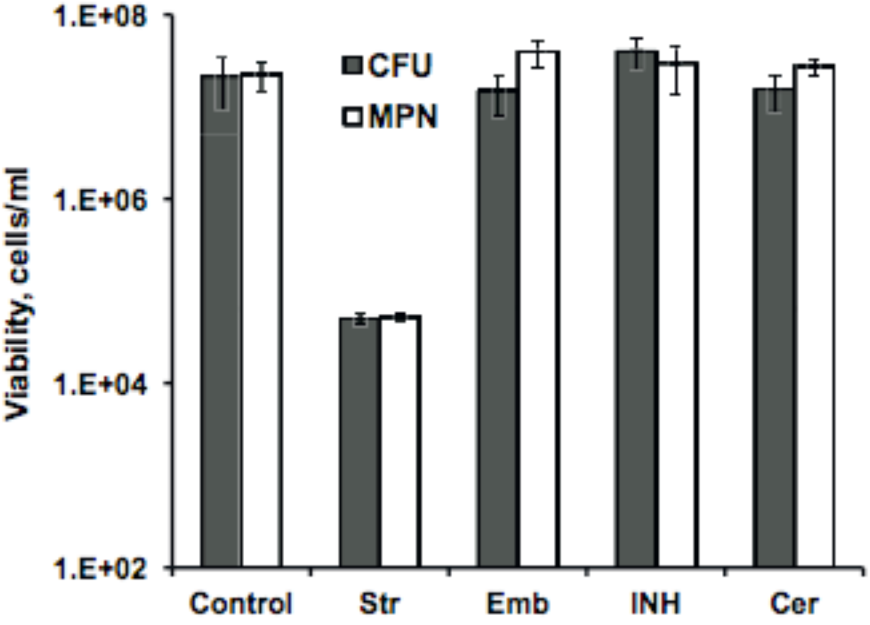

FIG S1 Effect of 24 h Antimicrobial treatment on the viability of stationary phase bacteria. 1-month old *M. bovis* BCG cultures were treated with antimicrobials and incubated for 24 h before assaying viability by measuring CFU and MPN. Average values for 3 independent experiments with standard deviations are shown. H_2O – water control; Emb- ethambutol; INH – isoniazid; Cer – cerulenin; Str – streptomycin. Only streptomycin had a significant bactericidal effect after 24 h exposure to stationary phase bacilli ($p<0.01$, t-test).

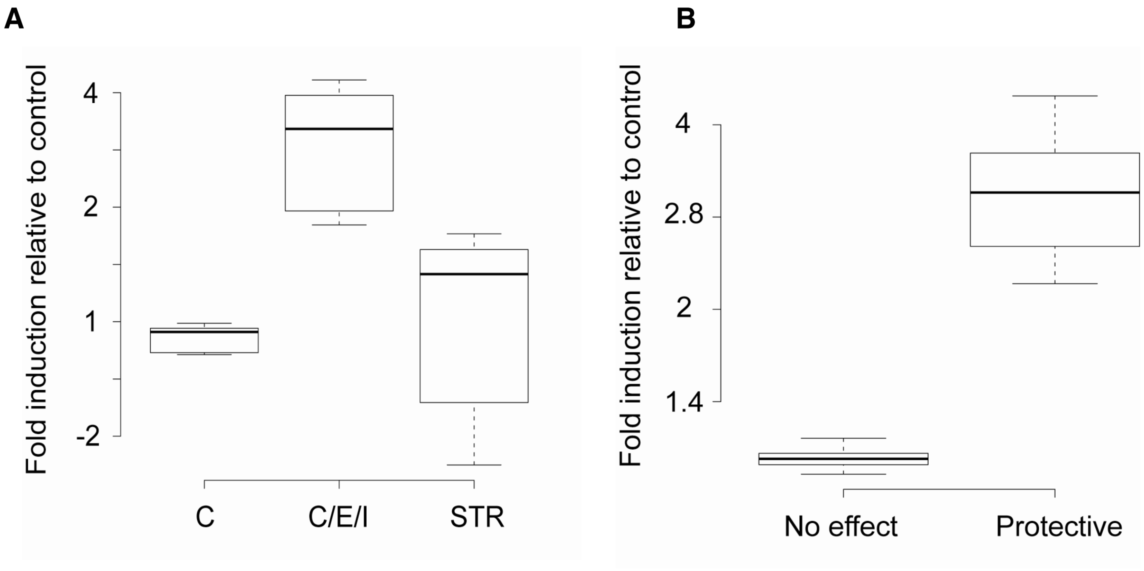

FIG S2 Box plots detailing genes that may be functionally important in prolonged stationary phase survival after antibiotic exposure, grouping conditions by protective effect at 2 months. **(A)** t-test (p-value <0.05, Benjamini and Hochberg multiple testing correction and fold change >2) identifying genes significantly induced after C/E/I exposure compared to both drug-free and STR-treated bacilli. C – Control, C/E/I (Cerulenin/Ethambutol/Isoniazid), STR – streptomycin. **(B)** ANOVA (p-value <0.05, Benjamini and Hochberg multiple testing correction and fold change >2) comparing treatments with a protective effect at 2 months (C/E/I) to treatments with no effect on long-term survival (C/STR). *Bcg1279c* (*raas*) was significantly induced in all samples treated with C/E/I.

pMind- *raas*$_{mtb}$ **pMind**

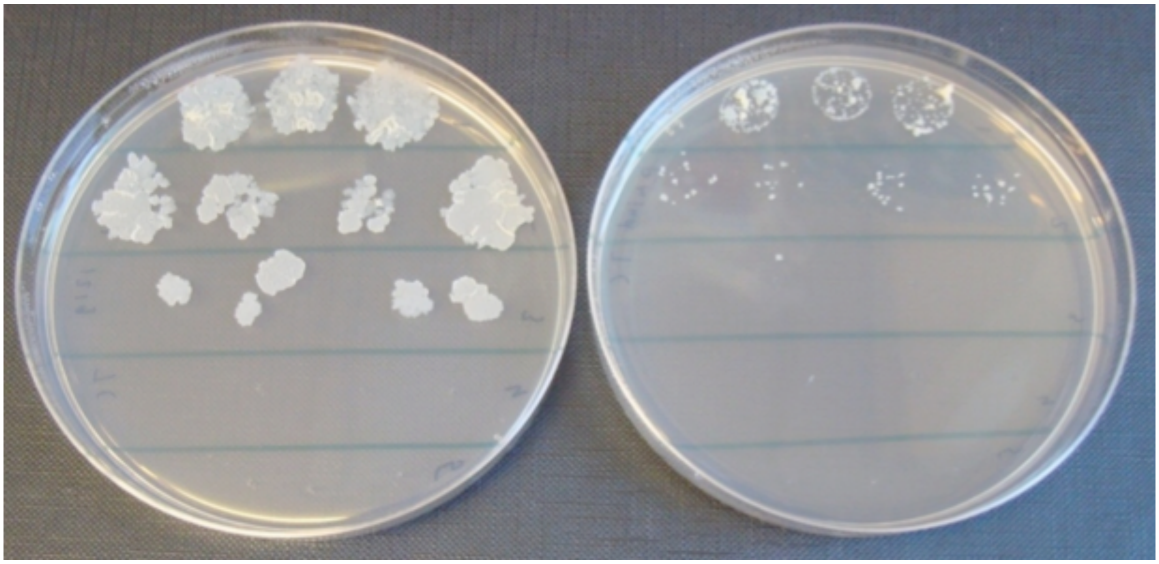

FIG S3 Effect of Raas over-expression on re-growth of *M. bovis* BCG. One month old pMind or pMind-*raas*$_{mtb}$ cells were serially diluted (10^{-1} to 10^{-5}), inoculated onto 7H10 agar plates and incubated for 4 weeks. The colony sizes reflect different dynamics of recovery, with *raas* over-expressing bacilli recovering more quickly than bacilli containing an empty vector.

A.

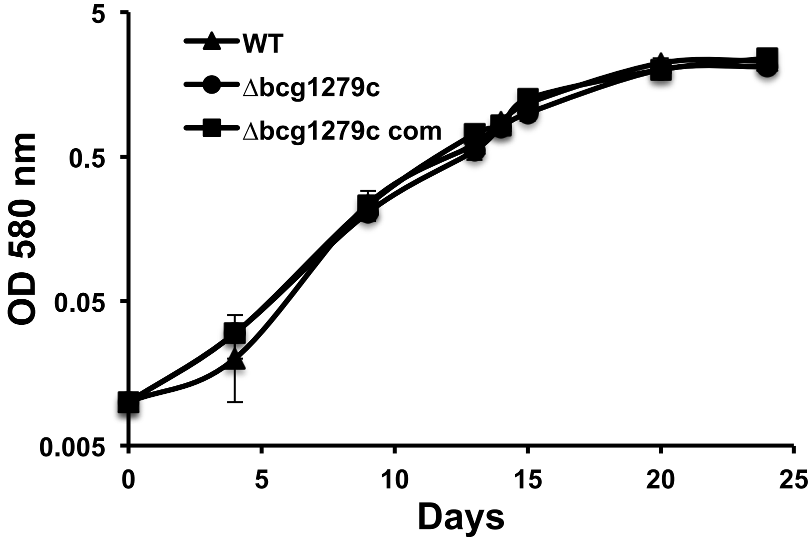

B.

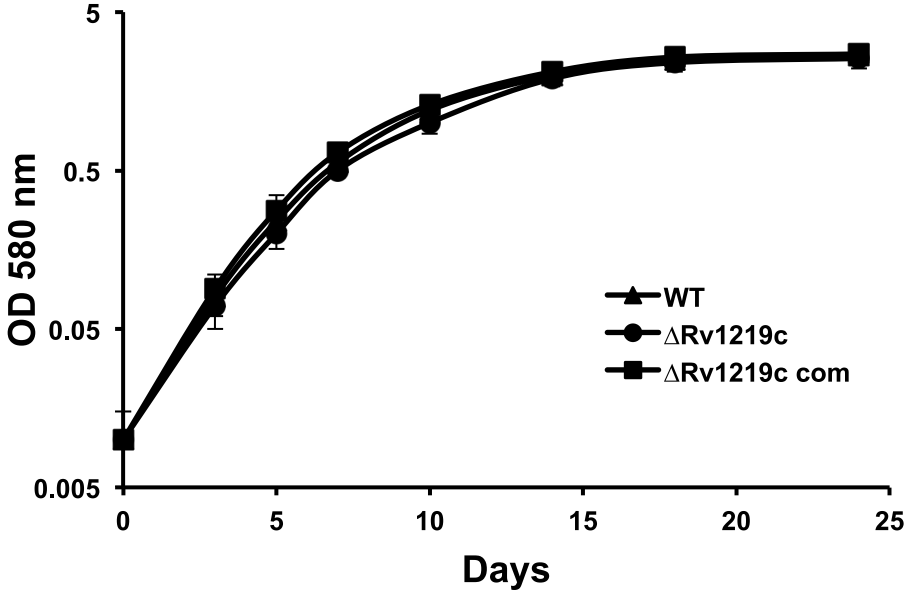

FIG S4 Growth of *M. bovis* BCG and *M. tuberculosis* Δ*raas* mutants *in vitro*. Mycobacterial strains were inoculated in supplemented Sauton's medium and incubated at 37°C with shaking (*M. tuberculosis*) or without shaking *M. bovis* BCG. Optical density was measured at 580 nm using a Jenway spectrophotometer.

Supplemental Experimental Procedures

Supplemented Sauton's medium

The following chemicals (g/litre) KH_2PO_4 0.5 g, $MgSO_4$ 0.5 g, asparagine 4 g, citric acid 2 g, ferric ammonium citrate 0.05 g were dissolved in Milli-Q water. Glycerol (1 ml) and 100 µl of 1 % (w/v) $ZnSO_4$ were added and pH was adjusted to 7.5 before autoclaving at 121°C for 15 min. Sterilised Sauton's medium was kept in dark place and used within 2 weeks. 900 ml of autoclaved Sauton's base were mixed with 100 ml of ADC supplement and 10 ml of Tween 80 10% (w/v) solution. ADC supplement contained (per 1 Litre) 50 g of Bovine serum albumin, 20 g of glucose and 8.5 g NaCl. ADC supplement and Tween 80 solutions were filter-sterilised.

Determination of Antimicrobial Susceptibility and Minimum Inhibitory Concentrations

To measure antibiotic sensitivity, serially diluted cells were spotted onto agar containing different concentrations of antimicrobials and the plates incubated at 37°C for up to 8 weeks, before counting colony forming units. Serially diluted cells were also inoculated into liquid medium containing drugs as a further measure of drug sensitivity. For isoniazid, ethambutol and reserpine, filter disc assays were also used. Briefly, 6 mm paper discs (Whatman), soaked with a range of antibiotic concentrations were applied to agar plates inoculated with a lawn of 10^7 *M. bovis* BCG cells. Plates were incubated at 37°C for 4 weeks, before measuring the diameters of the resultant inhibition zones.

For determination of MIC values, a micro-dilution method was used; $2x10^6$ *M. bovis* BCG cells were inoculated into 100 µl Sauton's or 7H9 supplemented medium containing different concentrations of antimicrobials. For ethambutol the range of concentrations was 0.1-5 µg/ml, for isoniazid - 0.01-1 µg/ml; for cerulenin - 0.1-20 µg/ml; for

streptomycin - 0.05-20 μg/ml. Microplates were sealed with Nescofilm and incubated at 37°C for 4 weeks without shaking. Mycobacterial growth was assessed visually and by measuring OD at 580nm in a Varioskan plate reader. The MIC corresponded to the first concentration of antimicrobial agent at which no visible growth was observed. For all *M. bovis* BCG strains tested, we determined the following MICs: ethambutol 0.8 μg/ml; isoniazid 0.02 μg/ml; cerulenin 1 μg/ml; rifampicin 0.1 μg/ml; streptomycin 1 μg/ml.

The presence of active ethambutol, isoniazid and streptomycin in culture supernatants after two months incubation was detected by their inhibitory effect on *M. bovis* BCG growth. Briefly, log phase *M. bovis* BCG cells were inoculated into 5ml of medium containing 50% (v/v) of culture supernatants from drug-free cultures or drug-treated cultures. Control supernatant from drug-free cultures in prolonged stationary phase did not prevent the growth of *M. bovis* BCG, while culture supernatants from drug-treated cultures inhibited growth as measured by CFU counting.

Transcriptional Profiling of Responses to Antimicrobial Exposure

Total RNA was isolated from 30 ml *M. bovis* BCG stationary phase cultures after 24 hours exposure to antimicrobial compounds using the Trizol method (1). RNA samples, extracted in duplicate or triplicate, were DNase-treated (Turbo DNA-free, Ambion) and purified using RNeasy columns (Qiagen). *M. tuberculosis* responses to drug treatment were profiled using a whole genome microarray, generated by the Bacterial Microarray Group at St. George's (Array Express accession number A-BUGS-23; http://bugs.sgul.ac.uk/A-BUGS-23). Hybridizations (3-7 per condition) were conducted as previously described, using *M. tuberculosis* genomic DNA as a common reference. The hybridized slides were scanned sequentially at 532 nm and 635 nm, corresponding to Cy3 and Cy5 excitation maxima, using an Affymetrix 428 Array Scanner (MWG).

Comparative spot intensities from the images were calculated using Imagene 5.5 (BioDiscovery) and imported into GeneSpring GX 7.3.1 (Agilent Technologies). The array data were normalized to the 50th percentile of all genes detected to be present on the array, and filtered to include only genes flagged to be present on 80% of the arrays. Significantly differentially expressed genes were identified in pair-wise comparisons between control and antibiotic-treated cells using a t-test (p-value <0.05 with Benjamini and Hochberg multiple testing correction) and a fold change >2. Five genes were significantly induced by cerulenin, and ethambutol and isoniazid relative to drug-free bacilli but were not induced by streptomycin exposure (Table S1). Similar clusters of genes (all including *Rv1219c*) were identified in alternative analysis strategies by grouping antibiotic treatments by protective effect after prolonged stationary phase (Figure S2).

Microarray data were confirmed by quantitative RT-PCR. DNA contamination was removed with Turbo DNA-free DNAase (Ambion) before cDNA was generated using Superscript Reverse Transcriptase II (Invitrogen) and gene-specific primers (Table S2). Q-PCR was performed in a Corbett Rotor Gene 6000 real time thermocycler using Absolute QPCR SYBR Green mix (Thermo) and gene-specific primers. Levels of expression were normalized to *16s rRNA* (2).

Transcriptomics of *Raas* Knockout and Complemented *M. bovis* BCG Strains

Mycobacterial RNA was extracted from WT, *raas* deleted and complemented *M. bovis* BCG strains using the GTC/Trizol method (3), then DNase-treated and purified using RNeasy columns (Qiagen). Microarray hybridizations were conducted as described above, hybridizing RNA derived from three biological replicates. Significantly differentially expressed genes were identified comparing knockout mutant to both WT

and complemented strains using a t-test (p-value <0.05 with Benjamini and Hochberg multiple testing correction) and a >2 fold change threshold (Table 1). Fully annotated microarray data have been deposited in B*μ*G@Sbase (accession number E-BUGS-123; http://bugs.sgul.ac.uk/E-BUGS-123) and also Array Express (accession number E-BUGS-123).

Supplemental References

1. **Betts, J.C., P. T. Lukey, L. C. Robb, R. A. McAdam and K. Duncan**. 2002. Evaluation of a nutrient starvation model of *Mycobacterium tuberculosis* persistence by gene and protein expression profiling. Mol. Microbiol. **43**: 717-731.
2. **Cheah, E.S. et al.** 2010. A two-tube combined TaqMan/SYBR Green assay to identify mycobacteria and detect single global lineage-defining polymorphisms in *Mycobacterium tuberculosis*. J. Mol. Diagn. **12**: 250-256.
3. **Waddell, S. J. and P. D. Butcher**. 2010. Use of DNA arrays to study transcriptional responses to antimycobacterial compounds, in Antibiotic Resistance Protocols (SH Gillespie and TD McHugh, eds.). Methods Mol. Biol. **642**:75-91

Table S1. Genes identified by microarray analysis to be significantly differentially expressed in *M. bovis* BCG in the presence of cell-wall targeting drugs (ethambutol and isoniazid and cerulenin) relative to genes in drug-free bacilli that are not up/down-regulated by streptomycin exposure. Fold change values comparing drug-treated to drug-free controls from microarray* and qRT-PCR** analyses are detailed. The table is ordered by direction of regulation and chromosome location. ND –not determined.

Gene	Predicted function (*Mtb* gene)	Ethambutol		Isoniazid		Cerulenin	
		Array	qRTPCR	Array	qRTPCR	Array	qRTPCR
mmpS5 (bcg0726c)	Conserved membrane protein	5.33	2.76	3.47	-4.46	4.36	2.99
bcg1278c	Tetronasin ABC transporter	2.36	-1.72	2.33	-4.55	7.86	9.70
bcg1279c	Transcriptional regulator	2.94	2.93	2.93	2.16	7.09	23.28
bcg2761c (clgR)	Transcriptional regulator	3.18	N/D	2.42	N/D	3.63	N/D
ethA (*bcg3917c*)	Monooxygenas e	4.07	-1.43	2.88	-1.79	1.99	4.05
pks6 a(b) (*bcg0443*)	Polyketide synthase	-3.76	-3.33	-3.44	-3.85	-4.04	-1.56
pyrG (bcg1737)	CTP synthase	-4.56	-2.86	-5.18	-2.94	-4.92	-1.56

*t-test p-value <0.05 with Benjamini and Hochberg multiple testing correction; **average values, the standard deviations for all genes did not exceed 10% of average values.

Table S2. Oligonucleotides used in the study

Primer	Sequence (5'-3')	Application
1219 F1	CGAATAGGATCCCGCCGAA AACGAAGAGTC	Amplification of upstream *raas* region for generation of $raas_{Mtb}$ and *raas* mutants; *Bam*HI site introduced.
1219 R1	GCCGAATCTAGAGCTCAAC AAGGAGAAGCA	Amplification of upstream *raas* region for generation of $raas_{Mtb}$ and *raas* mutants; *XbaI* site introduced.
1219F2	GGCCGCTCTAGAGATCCTG GCGTGCGCGGT	Amplification of downstream *raas* fragment for generation of $raas_{Mtb}$ and *raas* mutants; *Xba*I site introduced.
1219R2	TGCGACAAGCTTCTCACCG GCTAGCGACGC	Amplification of downstream *raas* fragment for generation of $raas_{Mtb}$ and *raas* mutants; *Hin*dIII site introduced
1219DF1	ACTCCGGCACTGGTGCCG	Diagnostic primers for confirmation of the *raas* deletion in $raas_{Mtb}$ and *raas* mutants
1219DR1	GCA CTT CGC CCT CGC GCA	Diagnostic primers for confirmation of the *raas* deletion in $raas_{Mtb}$ and *raas* mutants
Rv1219F6OE	ATCGGATCC CGGGGATGA ACGTACGTTTAA TAT	Amplification of $raas_{Mtb}$ for cloning in pMind; *Bam*HI site introduced
Rv1219R8OE	CCAACTAGTTCAGCCGAC ATGTGCTTCTCC TTG	Amplification of $raas_{Mtb}$ for cloning in pMind; *Spe*I site introduced
1219F4	ATCCTGAACATGCGTTCAGC CGATCTGACC	Diagnostic primers for $raas_{Mtb}$ and $raas_{bcg}$ deletion mutants
1219R4	CAA CCG CAC GCT TCC GCC GTC GGC CTT CAC	Diagnostic primers for $raas_{Mtb}$ and $raas_{bcg}$ deletion mutants
Rv1219F exint	GGTACTAGTATGCGTTCAGC CGATCTGACC	Amplification of ras_{Mtb} for cloning in pRBexint; *Spe*I site introduced
Rv1219R exint	ACAGTTAACTCAGCCGACAT GTGCTTCTCC	Amplification of $raas_{Mtb}$ for cloning in pRBexint; *Hpa*II site introduced
IntF1	TGAACCTGCGGCCTTCCGC T	Verification of integration of $raas_{Mtb\text{-}p}$RBexint
IntR1	GAACTCCGACGCGCACGAG C	Verification of integration of $raas_{Mtb\text{-}p}$RBexint
EthAFwd	ATGACCGAGCACCTCGACG TTGT	qRT-PCR
EthARev	GTAGCTCTTGGTCGGGCAA CGG	qRT-PCR
rv1218c Fwd	GTTCACTAGACGCCTTGCG GCA	qRT-PCR
rv1218c Rev	CGACTCCCTTGATTTGGCTG AGGT	qRT-PCR
rv1219c	AGGATCAGAGAGGCGGCCA	qRT-PCR

Fwd	TCGAA	
rv1219c Rev	ATGGTGGATGACCAATGCC GCGCTC	qRT-PCR
pks6 Fwd	ATGACAGACGGTTCGGTCA CTGCG	qRT-PCR
pks6 Rev	GGATGACACTCGATATGCG TGGACA	qRT-PCR
pyrG Fwd	GATTGGACCGAATGGGACG ACC	qRT-PCR
pyrG Rev	CGGATAATTCGACGTACTTG CCCAC	qRT-PCR
SigA Fwd	TTCGCGCCTACCTCAAACAG	qRT-PCR
SigA Rev	GCTAGCTCGACCTCTTCCTC G	qRT-PCR
RT1215F1	ACCTACGACATGGAGCAGA TGCAA	qRT-PCR
RT1215R1	TGTGCAAGTTGTTGTCCGAG AAGC	qRT-PCR
RT1216F1	AGGGCCGAACGATTCAGAA GTTCA	qRT-PCR
RT1216R1	GCACTGACGACCAACCATA ACGAT	qRT-PCR
RT1217F1	TGTACATCGCCAGCGTCGA AA	qRT-PCR
RT1217R1	AAACATCCCGGCTTTCCAGA TTCC	qRT-PCR
RT1218F1	AAGACCGTCGAAAGCGGTT CACTA	qRT-PCR
RT1218R1	TGAGTTCTCTCAGGCTTTCG CTGT	qRT-PCR
RT1219F1	CAG GAT CAG AGA GGC GGC CAT C GAA	qRT-PCR
RT1219R1	ATG GTC GAT GAC AAT GCC GCG CTC	qRT-PCR
RT-drrCF	ATTGGGTTTCCGGTTTCGAC AAGG	qRT-PCR
RT-drrCR	CCTTCGACAACAACGGTTTG TGCT	qRT-PCR
RT-3489F	TCTACCAAATCTGACCACGG CGAA	qRT-PCR
RT-3489R	TCCAATACCGAGCATGGAC AGGAA	qRT-PCR
RT-mmps5F1	TTTGGTTCCGAAGGCATCTT GGTG	qRT-PCR
RT mmps5R1	AAACTTCGTACTCCACCACC TTGG	qRT-PCR
MYCO16SF	GAA ACT GGGTCTAATACCG	qRT-PCR

MYCO16R	ATCTCAGTCCCAGTGTGG	qRT-PCR
1219prF3	GCA TGA TCT AGA ATTCGGCGAGCAGACGCA	EMSA
1219prR1	TCA ATG GGA TCC GTTCAGGATATTAAACGT	EMSA
Pr6F	GGGCGAGCAGACGCAAAAT CGCCCTG	EMSA
Pr6R	GGGCAGGGCGATTTTGCGT CTGCTCG	EMSA
Pr7	CAGACGCAAAATCGCCCTG AACCGTGCGTTCCAGGGCG ATTTTGCGTCTG	EMSA
Pr8f	GGGCGAGCAGACGCAAAAT CGCCCTGAACCGTGCG	EMSA
Pr8R	GGGCGCACGGTTCAGGGCG ATTTTGCGTCTGCTCG	EMSA
1219pr14F	GGGATGAACGTACGTTTAAT ATCCTGAACATGCGTTCAG	EMSA
1219pr14R	GGGCTGAACGCATGTTCAG GATATTAAACGTACGTTCAT	EMSA
1219pr15F	GGGCAAAGTTCGCGGCCTT CACGGATTCCCAA	EMSA
1219pr15R	GGGTTGGGAATCCGTGAAG GCCGCGAACTTTG	EMSA
1219pr16F	GGGATTCCCAACGGCGCCC TCCCTTGACCGGGGATG	EMSA
1219pr16R	GGGCATCCCCGGTCAAGGG AGGGCGCCGTTGGGAAT	EMSA
M5F	GGGATGAACGTACG**AAA**AA TATCCTGAACATGCGTTCAG	EMSA
M5R	GGGCTGAACGCATGTTCAG GATATTTTTCGTACGTTCAT	EMSA
M6F	GGGTGAACATGCGTTCA	EMSA
M6R	GGGTGAACGCATGTTCA	EMSA
1219pETF	TCA CAT ATG ATG CGT TCA GCC GAT CTG ACC	Generation a construct for the Raas recombinant protein
1219pETR	ACA GCTAGC TCA GCC GAC ATG TGC TTC TCC	Generation a construct for the Raas recombinant protein
